# Detailed classification of swimming paths in the Morris Water Maze: multiple strategies within one trial

**DOI:** 10.1038/srep14562

**Published:** 2015-10-01

**Authors:** Tiago V. Gehring, Gediminas Luksys, Carmen Sandi, Eleni Vasilaki

**Affiliations:** 1 Department of Computer Science, University of Sheffield, Sheffield, UK; 2 Division of Cognitive Neuroscience, University of Basel, Basel, Switzerland; 3 Laboratory of Behavioral Genetics, Brain Mind Institute, EPFL, Lausanne, Switzerland; 4 Theoretical Neurobiology and Neuroengineering Lab, University of Antwerp, Wilrijk, Belgium; 5 INSIGNEO Institute for in Silico Medicine, University of Sheffield, Sheffield, UK

## Abstract

The Morris Water Maze is a widely used task in studies of spatial learning with rodents. Classical performance measures of animals in the Morris Water Maze include the escape latency, and the cumulative distance to the platform. Other methods focus on classifying trajectory patterns to stereotypical classes representing different animal strategies. However, these approaches typically consider trajectories as a whole, and as a consequence they assign one full trajectory to one class, whereas animals often switch between these strategies, and their corresponding classes, within a single trial. To this end, we take a different approach: we look for segments of diverse animal behaviour within one trial and employ a semi-automated classification method for identifying the various strategies exhibited by the animals within a trial. Our method allows us to reveal significant and systematic differences in the exploration strategies of two animal groups (stressed, non-stressed), that would be unobserved by earlier methods.

The Morris Water Maze (MWM)^1^,^2^ navigation task is widely used in spatial learning studies^3^. In this task, rodents are placed in a circular water pool with the goal of finding a submerged escape platform, which is made invisible by using, for instance, milky water or completely black walls in the experimental setup. Because the platform is hidden, animals cannot memorise its location directly and have to rely on external contextual visual cues for orientation. The MWM pool sizes vary greatly between experiments and depend on the types of animals used. For experiments with rats typical maze dimensions are a tank with a diameter of 1 to 2 meters, in which a 10–15 cm platform is placed^4^,^5^. A typical experimental protocol consists of a set of trials, divided over a few days, during which the starting position of the animals is changed but the platform is kept at the same location. In a subsequent probe trial, the platform is removed and the animals’ memory is evaluated by their swimming persistence in the surroundings of the previous platform location^6^. This testing protocol is known as *spatial acquisition*^3^,^5^. Typically, the initial trials in the MWM are characterised by the animals’ tendency to spend most of the time next to the walls (a behaviour known as *thigmotaxis*^7^), or to perform random searches in the arena. In later training trials, however, animals show a gradual change in behaviour, characterised by progressive active searches for the platform. As they get familiar with the environment and testing rules, their ability of finding the platform from different starting positions is improved, as indicated by the time to reach the platform.

Despite its widespread use in rodent behavioural studies, many studies quantify the behaviour in the MWM and base their results on just a handful of simple direct measures, without taking the different behavioural patterns into account. One of the most commonly used performance measures is the *escape latency*, defined as the time for the animal to find the platform and escape the maze. Other measures proposed in the literature include the swimming path length, which is suggested to be a better measure than escape latency^1^,^8^,^9^.

It has long been recognised, however, that these simple measures alone are not sufficient to quantify the wide range of different behaviours observed in the experiments^10^,^11^. In order to be able to better characterise the behaviour in the MWM, more sophisticated quantification methods were proposed over the years. Petrosini *et al.*^12^, for example, developed a scoring system of swimming paths based on measures such as the time that the animal spends in the “right” or “wrong” quadrants of the maze. Dalm *et al.*^10^, showed that the cumulative distance to the platform is correlated with the time spent next to the platform, but not with the escape latency. By combining measures, they were able to create a classification method for swimming paths, assigning each one of them to one of three different classes of behaviour. Their method, however, was applied only to a small group of animals (24) and quantified the behaviour of swimming paths of approximately the same length only in the first two trials; they did not consider later stages of learning.

Categorisation methods of swimming paths in the MWM were also proposed in other studies. Wolfer and Lipp^13^, for example, associated various types of behaviour with different stages of learning. They also computed more than a dozen measures for each swimming path and showed that these can be used to classify the paths into different behavioural classes. However, they noted that their categorisation is valid only for large populations of animals and that single individuals might skip learning stages, or display more than one type of behaviour within a single trial.

Graziano *et al.*^14^ developed a more complete classification of swimming paths by dividing them into seven different classes, or *exploration strategies* in their terminology. The classes of behaviour ranged from thigmotaxis, where the animal almost never finds the platform (a type of behaviour mostly seen in early trials), to straight paths to the platform (*direct finding*). These classes showed a high correlation with the escape latency value but offered a more detailed quantification of the different stages of learning. By using the escape latency in addition to more than two dozen other path measures, they were able to create an automatic classifier that could assign swimming paths to one exploration strategy. Similar to previous attempts, however, their method was focused on assigning each swimming path to a single exploration strategy. The method is therefore not well suited to quantify mixed types of behaviour within the same trial, which frequently characterise animals’ strategies in the maze. In particular, longer swimming paths frequently show traits of more than one exploration strategy, making an unambiguous classification more difficult.

In this work a new, more granular classification method for swimming paths in the MWM is presented. In order to be able to quantify changes in behaviour within a trial, the classification is targeted not at complete swimming paths, but rather at shorter path segments. Our method consists of classifying multiple segments of a single swimming path into stereotypical classes of behaviour. As a result, swimming paths are not mapped to a single behavioural class, but to several classes. This approach allows us to identify subtle changes in behaviour between trials and among different groups of animals.

The development of this new method was motivated by the necessity of a more sophisticated quantification framework for analysing MWM data. The method was applied to a set of behavioural data where a strong manipulation (peripubertal stress) did not yield obvious learning performance differences compared to a control group when using more traditional quantification methods. A manual classification of the full swimming paths also didn’t produce satisfactory results because many of them showed mixed types of behaviour and the data set was not sufficiently large (around 300 paths per group). This led to large uncertainties in the classification making clear that a new approach was needed.

The segmentation of swimming paths we propose here is performed automatically by custom analysis tools, and is done so that each segment has approximately the same length and overlaps substantially with the previous ones. This overlap is important to make sure that the classification is not affected by an unfavourable segmentation, but it also means that a large number of segments (from a couple of dozen to a few hundred per swimming path) are generated. The large number of path segments (up to 30,000 for the data set considered here) makes a complete manual classification intractable for all practical purposes. In order to overcome this problem, a semi-automatic classification method is adopted. The classification method, based on a *semi-supervised* class of machine learning algorithms, is able to automatically classify segments into behavioural classes based on a small percentage (between 5% and 12% of the segments in our case) of manually classified data. Because the classifier is based on a data clustering algorithm, it is also ideally suited for finding patterns in data, so that behaviour classes don’t need to be known *a priori*.

The custom set of software tools we developed includes a Graphical User Interface (GUI) which can be used to interactively segment, label, classify, and visualise the classification results of swimming paths. All code developed is freely available in ModelDB and in Git repository (https://bitbucket.org/tiagogehring/mwm_trajectories).

In what follows, we first introduce the classification procedure. Our method is then applied to our data set in order to demonstrate that it can indeed successfully identify differences in behaviour, where traditional analyses based on individual path measures fail. We then discuss results and future work perspectives. Finally, we present a detailed description of the proposed methods.

## Results

**Figure 1:**
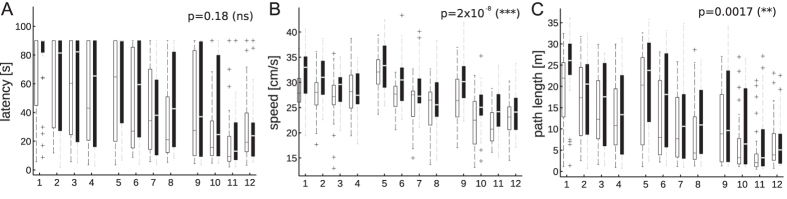
Comparison of full trajectory metrics for two groups of animals over a set of 12 trials. *White (black) boxes*: control (stress) group. Boxes represent the first, second (median, shown as a band) and third quartiles; whiskers are the minimum and maximum values. Outliers are marked as a cross. The p-values of a Friedman non-parametric test comparing both groups of animals over the full set of trials is shown in each plot (see Methods for a discussion and interpretation of the Friedman test and the p-values). (**A**) Escape latency shows a wide dispersion of values until later trials. (**B**) Average movement speed shows that stressed animals move substantially faster than non-stressed ones. (**C**) Average path length. Stressed animals tend to sweep longer paths than control animals; the difference between the two groups is however less distinctive than when comparing the movement speeds.

We suggest a new classification method for swimming trajectories that we apply, as a case study, on a set of 57 rats, 30 of which were submitted to peripubertal stress^15^,^16^,^17^; the other 27 animals were the control group. The swimming paths of a set of 12 trials, divided into three daily sessions over three consecutive days, were acquired at the Laboratory of Behavioural Genetics, EPFL, Switzerland. Commonly used behavioural measures of learning, such as escape latency (Fig. 1A), fail to show significant differences between the two groups. Although differences in movement speed are pronounced (Fig. 1B), they cannot be unambiguously interpreted as differences in learning. The technique we present here, on the other hand, is able to capture systematic and significant differences between the two groups.

### Method validation

In order to validate our classification method, the initial classification results were compared to manually tagged swimming paths. The manual tagging consists of adding one or more labels to each path, depending on which behavioural traits were visually identifiable. This manual labelling (supplementary Fig. S2) was limited to four major behavioural classes easily identifiable by quick inspection: thigmotaxis, scanning of the region around the platform (called *target scanning* here), *incursion*, or paths where the animal still touches the walls but start moving inwards, and *scanning*, paths where the more central regions of the arena are searched. We classified the swimming paths by our method and compared the results with the manual labels in order to test the quality of the classification procedure.

**Figure 2:**
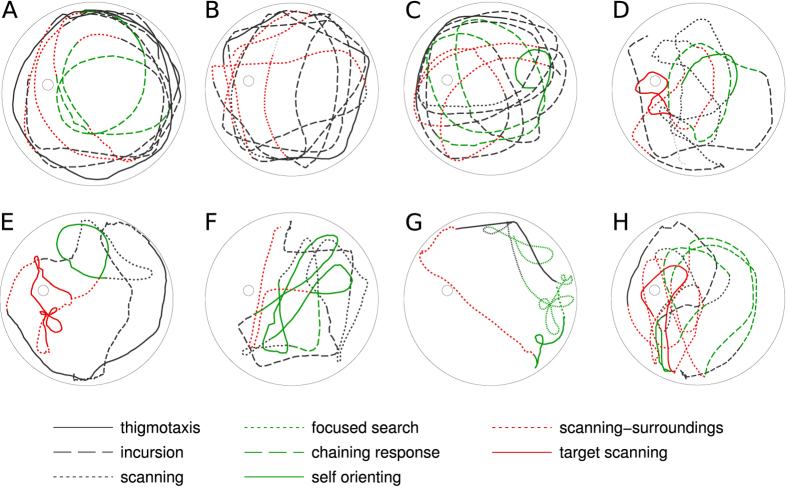
Examples of swimming paths showing different types of behaviour. Data: Laboratory of Behavioural Genetics, EPFL. Swimming paths were segmented and the generated segments were classified into a total of eight different types of behaviour, distinguishable by different line types/colours. Behavioural classes: (**i**) *Thigmotaxis* (solid-black lines): Time is spent almost exclusively next to the walls; (**ii**) *Incursion* (dashed-black lines): paths where animal still touches the walls but starts making incursions inwards; (**iii**) *Scanning* (dotted-black lines): characterised by tighter paths that sweep a specific region of the arena; (**iv**) *Focused search* (dotted-green lines): animal randomly searches a very small area of the arena; (**v**) *Chaining response* (dashed-green lines): concentric paths where the animal memorises the distance from the walls to the platform^13^; (**vi**) *Self-orienting* (solid-green lines): Paths where the animal makes one full turn to orient himself^14^; (**vii**) *Scanning-surroundings* (dotted-red lines): Open paths passing through a critical region around the platform; (**viii**) *Target scanning* (solid-red lines): search is focused on regions next to or surrounding the platform.

Our method produces results that offer a detailed overview of the swimming paths and are able to identify four additional types of behaviour that were not included in the manual labelling of swimming paths. Those were: *chaining-response*^13^, characterised by concentric paths where the animal sweeps all the points at the platform distance from the wall; *focused-search*, where the animal limits its search to very small areas randomly and repeatedly sweeping them; *self-orienting*^14^, characterised by a loop where the animal orients itself in the arena; and *scanning-surroundings*, or paths that cross a critical region around the arena but are not limited to this region. Figure 2 shows examples of swimming paths including all eight behavioural classes, as identified by our classification method.

The choice of classes was motivated by stereotypical behaviours that were observed during the classification and by the objective to distinguish subtle behavioural differences that may be crucial for the learning outcome. For this reason, a distinction was made between paths that pass nearby the platform but are not centred on it (scanning-surroundings) and closed paths that circle the platform and where the animal actively searches for it (target-scanning). A distinction was also made between paths concentrated almost exclusively on the periphery of the platform (thigmotaxis) and ones in which the animal starts moving inwards (incursion). This allowed for a more gradual characterisation of the behavioural changes, which would be difficult to achieve with previous classification methods.

Self-orienting, a behaviour described in Graziano *et al.*^14^, and chaining response^13^, where the animal memorises the distance from the walls to the platform, are intermediate phases of spatial learning frequently observed in the experiments and were, therefore, assigned to individual classes. Finally, scanning and focused-search are both types of behaviour associated with random searches of other regions of the arena, mainly the centre in the case of scanning. The difference between these two cases is that in focused-search the search is limited to a very small region, whereas in scanning larger areas are swept. Given their difference, these trajectories were split into separate classes, but are otherwise similar, in the sense that the search does not target the platform but rather other parts of the arena.

According to our classification method, we first split the trajectories into multiple segments of constant length, where each segment of the trajectory overlapped with the previous one by a fixed amount. The classification was done for segments of 250 cm with an overlap of 70% and 90% and for segments of 300 cm with a 70% overlap. During the segmentation process, a number of segments between 8,000 and 30,000 were generated. As a further consistency check, the classification was done multiple times; the different classifications were then compared and combined (see Methods) for producing the final results.

**Figure 3:**
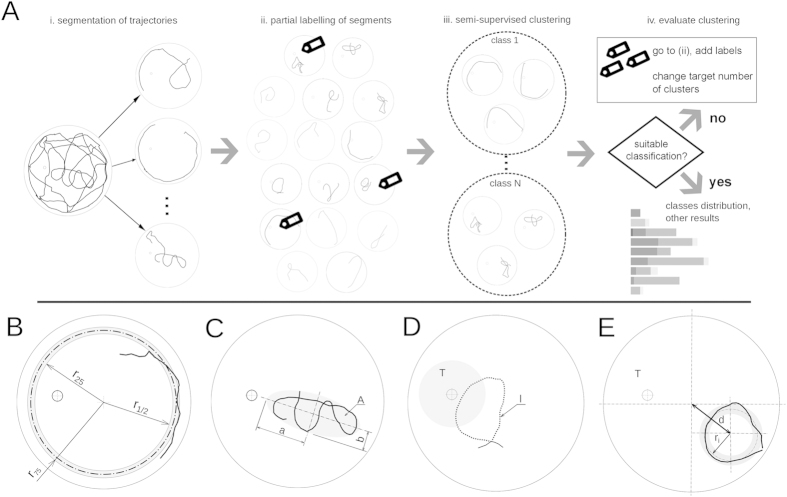
(**A**) Diagram illustrating the swimming path classification method. Swimming paths are first segmented and then classified by means of a semi-supervised clustering algorithm. (**B**–**E**) Definition of variables used for computing the measures, or feature values, for each swimming path segment. The features are an essential part of the clustering process since the feature values are used to estimate how similar the different segments are.

Swimming path segments, generated in the partitioning of the full trajectories, were mapped to one of the eight behavioural classes by means of a clustering algorithm (Fig. 3A). The clustering was done based on a set of eight features that were computed for each path segment (Table 1). A subset of the segments was also manually labelled to constrain and guide the clustering process, to map clusters to behavioural classes, and to validate the clustering results. The labelling of segments was done interactively, via custom software tools, which we have made publicly available (described in the supplementary information).

The labelling, clustering, and validation process was repeated until the major parts (at least 90% of the total length) of the swimming paths were covered by segments of known classes. Due to the overlap between the segments not all segments had to be classified in order to fully classify the swimming paths and thus clusters of segments with insufficient or inconsistent labelled data were discarded from the analysis. The percentage of the length of swimming paths were successfully classified was here defined as the *coverage* value.

During the clustering process different numbers of clusters were chosen and the ones giving the highest coverage value (i.e. fewer unclassified trajectory segments) were selected for evaluating the final results. The clustering algorithm was used a second time and clusters were further divided in an attempt to further improve the classification results (see Methods). This second stage not only improved the classification results, but also reduced the importance of initially selecting the “right” target number of clusters for the data set.

Parameters and results for the three classifications performed here are listed in Table 2. The percentage of corresponding segments that were assigned to the same class, or the consistency rate between both classifications, is shown in the last row of the table. Note that although the agreement factor seems to indicate an error of 10 to almost 30%, the differences are mostly due to segments that show traits of two different classes of behaviour and were mapped to opposite classes in both cases. These differences do not impact the final classification results significantly, because when computing the resulting class for a given point of a swimming path, all segments that overlap the point are taken into consideration. This means that small discrepancies of the actual segment classes in the case of transition segments average out, or that when there is a difference in the results, it is limited to only short path segments, of the order of one or two minimum discrete path length intervals (between 25 and 75 cm depending on the segmentation parameters).

After classifying the path segments, the distribution or evolution of strategies for each swimming path was computed. This was done by computing the resulting behavioural class for each minimum discrete path interval (25–90 cm depending on the segmentation). The class for each interval was selected based on the classes of the overlapping segments. Finally, the results of the classification were compared with the manually assigned labels for the four major classes of behaviour (thigmotaxis, incursion, scanning, and scanning target) assigned to the full swimming paths. We consider results correct if all manually assigned classes are detected by the classifier. The error rate computed in this way was found to be smaller than 3%, which showed that the semi-automatic classification is consistent with the manual classification.

### Detailed classification of swimming paths

**Figure 4:**
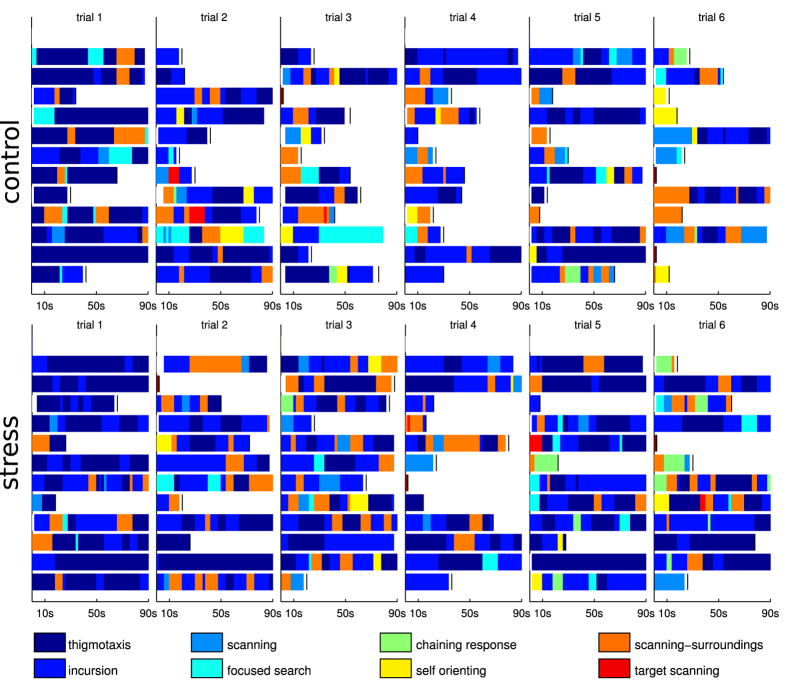
Classification results for the 6 first trials and 12 animals from the control (top) and stress (bottom) group. Each bar represents a full trial (up to 90 seconds) and shows changes in exploration strategies over the trial. Short paths, where the animal found the platform directly, and which were not segmented, are marked in dark red. White boxes indicate segments with behaviour not falling into any of the classes and which could not be categorised. The results show that paths almost always correspond to multiple types of behaviour. Also, it can be seen that on later trials animals are not only able to find the platform faster, but they also change their strategies.

Figure 4 shows the classification results for the first 6 trials and 12 animals each from the stress and control groups (for the complete plots including all the animals see supplementary Fig. S4). The detailed overview of each swimming path cannot be achieved with previous classification methods. The results show gradual changes in behaviour over the trials, and indicate at which points in time during a trial a given strategy was adopted. Multiple strategies are usually present within a single trial.

Initially, behaviours such as thigmotaxis and incursion dominate the trials, as the animals look for wall contact where they presumably feel safer. However, already at the second or third trials we observe an increased presence of the other more sophisticated strategies, where the animals explore more central regions of the arena (scanning), orient themselves (self-orienting) or actively look for the platform (chaining response, surroundings/target scanning).

### Application: comparison of stressed and non-stressed groups of animals

The classification method proposed here was used to look for differences in behaviour between two groups of animals. In the first group, animals were subjected to peripubertal stress. The second group is the control group. Basic analysis methods based on simple measures such as the escape latency (Fig. 1A) fail to show significant differences between the two groups of animals. One noticeable difference, however, is that stressed animals tend to move faster than non-stressed ones; their paths to platform are also longer (Fig. 1B–C).

The higher movement speeds of the stressed animals would suggest lower escape latencies since, other behaviour characteristics being equal, they would find the platform by chance more often. This, however, is not observed (Fig. 1A). In order to better understand and characterise the differences between the two groups of animals, the swimming paths were classified according to the semi-automatic method we propose.

**Figure 5:**
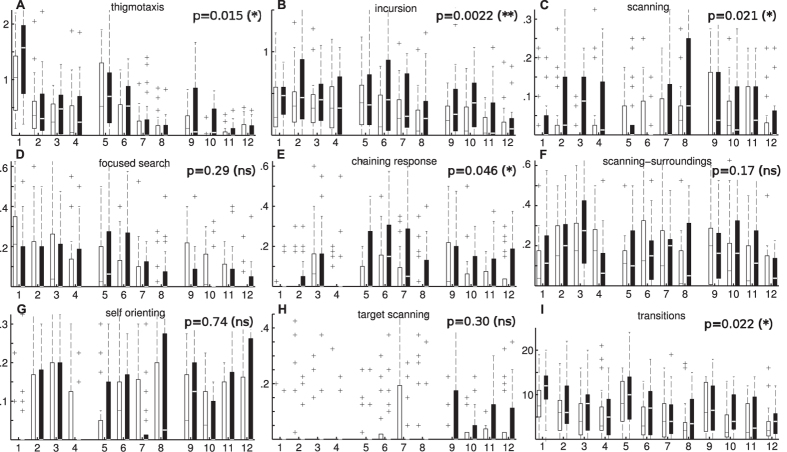
(**A**–**H**) Average segment lengths for each strategy adopted by stress (black) and control group (white) of animals for a set of 12 trials divided in 3 sessions (days). Plots show the average length in meters that animals spent in one strategy during each trial. Bars represent the first and third quartiles of the data; line shows the median and crosses the outliers. Whiskers (when shown) indicate minimum and maximum values. A Friedman test was used to compare both groups of animals over the complete set of trials; p-values are shown on the top right (see Methods for a discussion of the Friedman test and p-values). The results show that, as expected, stressed animals display longer average paths much more often but the increase is non-uniform among the different strategies. According to the plots there is a clear difference in the path lengths for the thigmotaxis, incursion, and scanning strategies, all of which are characterised by low chance of finding the platform. For the scanning-surroundings, self-orienting, and target scanning strategies, all which are associated with an increased chance of finding the platform, no statistically significant differences were found. Chaining response shows a slight difference in favour of the stress group; focused-search shows no significant differences. These results may explain why stressed animals sweep longer paths but on average they don’t find the platform faster that non-stressed animals (Fig. 1). (**I**) Number of transitions between strategies for both groups showing that stressed animals change their behaviour more often within single trials.

Figure 5 shows the distribution of strategies, using the same eight classes defined above, for both groups of animals over 12 trials. The plots show the average path lengths spent on one strategy over a trial. As expected from the differences in speed between both groups (Fig. 1B), stressed animals generally show longer paths than the control group. However, the difference in path lengths between the two groups is not homogeneous among all strategies. The results show that for the stressed animals there is a significant increase in thigmotaxis, incursion, and scanning classes, all associated with low chances of finding the platform (because most of the time is spent next to the walls or centre of the arena in these behaviour patterns). Other, more cognitively sophisticated strategies, such as self-orienting and target scanning, don’t show significant differences among the two groups. The exception is the chaining-response class, which shows a slight increase in the stress group. This difference, however, is small compared to the differences between stress and control animals in the first group of classes (i.e. thigmotaxis, incursion, and scanning).

Our analysis suggests that, although stressed animals sweep significantly longer paths, most of that difference can be attributed to staying near the walls and using simple exploration strategies such as scanning. More sophisticated strategies, where animals actively look for the platform, are used similarly by both experimental groups, explaining why stressed animals move faster but take about the same time (or even longer) to escape the maze.

Table 3 presents the results from another perspective: it compares the transition probabilities within the same trial between strategies for both the control (top table) and stress (bottom table) groups of animals. Values that deviate appreciably between both groups are shown in bold. It can be seen that stressed animals show a higher tendency for changing to thigmotaxis and incursion strategies, whereas the control group shows higher values for a transition to self-orienting and scanning surroundings strategies, both of which lead to increased chances of finding the platform. These results are in agreement with our conclusions above. Furthermore, they provide an alternative angle to look at the behaviour of both groups of animals, which would have been impossible using previous classification methods.

Finally, Fig. 5I shows that stressed animals also change strategies significantly more often than non-stressed ones within a single trial. It has been suggested^18^,^19^,^20^ that high levels of arousal or stress lead to labile attention and frequent strategy switches that may prevent efficient learning. In the reinforcement learning framework, which is relevant for computational modelling of the Morris Water Maze^21^,^22^, it has been shown^18^,^19^ that stress and anxiety can lead to steeper reward discounting, which may be the computational reason behind excessive strategy switches and impaired ability to learn tasks with significantly delayed rewards. Our results based on strategy classification provide empirical evidence to support this view.

## Discussion

The classification of swimming paths in the Morris Water Maze into behavioural classes is a useful method to study spatial learning in rodents, since the different classes of behaviour can be mapped to different stages of learning^6^. However, for some experiments, especially the ones with a limited number of animals, longer trials, or larger arena sizes, the discretization of all swimming paths into only a few behavioural classes might not be adequate. This is due to many swimming paths displaying characteristics of more than one class of behaviour; these trajectories cannot therefore be reliably assigned to a single class of behaviour^13^. This in effect means that results are valid only in a statistical sense, or for larger number of swimming paths. In order to address these limitations, we propose a new, more granular classification method that allows a detailed description of all strategies employed by the animals in a single trial.

Contrary to previous approaches, the main target of the classification method introduced here is not full swimming paths, which can vary greatly in length and consist of multiple types of behaviour, but rather shorter path segments of constant length. By classifying multiple segments of swimming paths, changes in behaviour within a single trial can be detected and quantified. As a result, instead of one single behavioural class, a distribution of classes is assigned to each swimming path. We showed that such detailed quantification can reveal subtle and novel behavioural differences between two groups of animals (Fig. 5); approaches comparing only individual path measures failed to provide clear insight. In addition, our method not only provides information about which types of behaviour were exhibited, but also at which point during the trial they were adopted (Fig. 4).

Our work provides a semi-automated classification method that requires only a reduced set of labelled data to map path segments to classes of behaviour. This is a useful feature, since it is virtually impossible to fully label large number of segments. Earlier work by Graziano *et al.*^14^ has also proposed an automated classification method of swimming paths. Their method was based on linear discriminant analysis (LDA), and made use of a high-dimensional feature space (more than 20 features), which made their classifier very robust. In contrast to our work, however, their method required a priori defined classes of behaviour, and classified complete swimming paths. Instead, we approached this problem by choosing to apply a semi-supervised clustering algorithm over a smaller feature space. The choice of the clustering algorithm was motivated by the fact that clustering is ideally suited for finding structure in data, without a priori knowledge of the classes. Therefore, behavioural classes do not have to be predefined, but can rather be identified by looking at common characteristics of elements of individual clusters. Also, clusters containing ambiguous segments (for example transitional segments between two classes) can easily be discarded.

In our implementation, we chose a set of features that captured both geometrical (focus, eccentricity, distance to centre spread, maximum loop length, inner radius variation) and positional (median distance to centre, target proximity, central displacement) aspects of the segments without adding redundancy between them. A smaller set of features was preferred because more features tend to separate the data into more clusters, and this in turn means that more labelled data has to be provided to map these clusters into respective classes.

Standard clustering algorithms usually fall in the unsupervised class of algorithms and find patterns in data without any previous knowledge. However, here we used a semi-supervised approach, where we could make use of a partial set of labelled data to guide the clustering process and improve the results. The labelled data are also essential to map clusters to classes and to compute an error estimate. The number of required labelled segments for classifying a set of swimming paths will depend on the specific data set at hand, on the number of classes and on parameters such as the minimum number of labels per cluster (see Methods). However, the labelling and clustering process can be performed incrementally, and so the number of labels can be increased until a suitable classification is found. To help with this process a custom Graphical User Interface (GUI) was developed here (supplementary information). This GUI makes it easy to label swimming paths or segments, to cluster the data, and to visualise the results of the clustering. All the code developed here is freely available in ModelDB (Access code: 185090).

One valid criticism of the classification methods of swimming paths is that the choice of measures can be very subjective. In his work, Korz^23^ makes this point and provides an alternative, bottom-up, approach for the analysis of swimming paths. His method is based on normalising the trajectories by reducing them to 50 equidistant points and using the actual coordinates of the points to compare trajectories. This is done by using principal component analysis (PCA), and has the advantage that no arbitrary measures have to be introduced. He also shows that the first few principal components are sufficient to account for most of the variability in the swimming paths, and can therefore be used to simplify trajectories. Although PCA-based methods have disadvantages such as interpretability of the principal components, in principle a similar approach could be used here for swimming path segments, if the intention were to avoid a subjective choice of features. In this case, the feature set could be defined by the first principal components of the segments rather than the geometrical and positional measures we used.

The work we present here can serve as a basis for defining more sophisticated scoring mechanisms for swimming paths. Scoring methods of swimming paths in the MWM were introduced in previous studies (e.g.^12^) and are important because they make it easy to compare swimming paths according to certain criteria between different groups of animals. The basis for such scoring system strategies could, for example, be a weighted sum of the relative distribution of strategies where their correlation with efficient swimming paths is used as a weighting factor. This approach, however, was not pursued here and was left for further investigations.

Finally, we note that, while the methods presented here were applied to trajectories of rats in the MWM, our method is likely to work similarly well for other species of rodents, such as mice. As mice and rats exhibit comparable trajectories in MWM, even if they learn at different speeds and possibly use slightly different strategies, the method itself should remain applicable, even if the exact number of behavioural classes and their specific description may differ. Our method is also general enough and can be easily applied to other behavioural tasks that employ different types of mazes and trajectories.

## Methods

### Morris Water Maze Experiments

Experiments were performed at the Laboratory of Behavioural Genetics, EPFL. All procedures were conducted in conformity with the Swiss National Institutional Guidelines on Animal Experimentation and approved by a license from the Swiss Cantonal Veterinary Office Committee for Animal Experimentation.

The water maze had a diameter of 2*m* with a submerged platform 12 cm in diameter. Recordings were performed by using an object tracking software, EthoVision^24^ version 3.1, and were done for a group of 57 rats over 12 trials. For 30 of the rats stress was induced at peripubertal age^15^; the other 27 were the control group. The trials were divided into 3 consecutive days, i.e., 4 trials per day; each day trials for each animal were applied consecutively, so that the inter-trial interval between the same day trials was only a few minutes. Starting position of the animals was alternated between trials. Animals were allowed to swim for 90 seconds and were guided to the platform if they failed to find it during this time interval.

### Data Analysis

Compared to previous approaches that attempted to categorise the behaviour of animals, our approach differs in first dividing the trajectories into segments. These segments, and not the full trajectories, are then classified into different classes of behaviour. Results from this analysis lead to a more detailed categorisation of swimming paths and allows for detection of mixed strategies within one trial.

Because the segmentation of swimming paths leads to a large number of segments to be classified, labelling all of them manually becomes intractable. To deal with this problem, a *semi-supervised* learning algorithm was used here for the classification of the segments. Such an algorithm requires only a small set of manually labelled data in order to constrain and validate the classification results. We performed a cross-validation procedure, in which part of the data was left out from the label set and was used instead to compare the results after the clustering (a 10-fold cross-validation was adopted here). The classification steps are shown in Fig. 3A and described below.

#### Segmentation of trajectories

The segmentation of trajectories is a method proposed to overcome the inherent difficulty of classifying very long swimming paths exhibiting many different types of behaviour. Classifying segments of trajectories is simpler because shorter paths usually don’t show multiple types of behaviour. Also, due to the segments having approximately the same length, the variance of feature values is smaller, which also improves the classification. The use of shorter segments and not the full trajectories for the classification therefore provides a precise and detailed quantification of swimming paths.

The segmentation adopted here (for a formal description see supplementary information) consists of dividing a trajectory into *N* segments of length *d* (with small variations due to the discrete nature and spacing of available data points) which overlap significantly with previous segments to reduce the classification variance due to unfavourable segmentations. The choice of the appropriate segment length depends on the size of the arena and on the stereotypical behaviour types of interest. The classification performance is not affected by small variations in the segment length, provided that the features are only weakly correlated with the length. This allows for mixing of segments of slightly different lengths in one classification. It also follows from this that the number of segments, which is related to the segment length and overlap used in the classification, does not have a large influence on the final classification results.

For the data here, recorded on an arena with a diameter of 2 meters, segment lengths of 250 cm and 300 cm and overlaps of 70% and 90% were adopted.

#### Computation of features

A set of eight features was selected for the classification procedure. Features are listed in Table 1 and a schematic overview of the required measures and definitions is shown in Fig. 3B–E. The features quantify different geometrical properties of the segments and their relative proximity to the platform. Where possible, they were made independent from the segment length. This way, as mentioned in the previous Section, we prevent small segment length variations from having an impact on the classification.

##### Median distance to centre

The average distance to the centre of the arena is a very useful measure to indicate if an animal spends most of its time next to the walls or moves also to more central parts of the arena; this value can be seen as a generalisation of other measures adopted in previous studies, such as the time spent next to the walls or at the centre of the arena^14^. This value is calculated from the trajectory by computing the distance to the centre of the arena for each data sample and average over the data. Instead of using the standard mean, however, the median radius will be considered here. The reason for using the median and not the mean is that the former is a more robust statistic, in the sense that a few points that are very distant from the others, such as ones at the edges of the segments, will have a smaller effect on the final value.

##### Interquartile range of the distance to centre

Besides the median of the radial distance, its spread can provide information about the type of behaviour involved. As a measure of spread, the interquartile range, or the distance between the first and last quartile of the data set (Fig. 3B), was considered. This value is again more robust than the more commonly used standard deviation.

##### Focus

The focus measures if and by how much the animal targets its search to specific parts of the arena. It is defined as *f* ≡1 − 4*A*/*πd*^2^, where *A* is the area of the minimum enclosing ellipse around the trajectory (Fig. 3C) and *d* is the segment length. For computing the enclosing ellipse the algorithm described in Moshtagh^25^ was used. With this definition a focus of 0 means that the segment is a perfectly circular path; larger focus values give an indication of increasingly closed paths.

##### Target proximity

The proximity value measures the percentage of the path lying within a circle centred at the platform (Fig. 3D) and with a radius of 6 times the platform radius.

##### Eccentricity

The eccentricity measures how elongated are the paths; it also makes use of the values computed from the minimum enclosing ellipsoid. It is defined as 
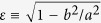
, where *a* and *b* are semi-major and semi-minor axis of the enclosing ellipse (Fig. 3C).

##### Maximum loop length

This value measures the length of the longest loop, or self-intersecting sub-segment of the path. To compute this value all pairs of lines defined by two consecutive trajectory points were tested for intersection. If no intersection was present a value of zero was assigned to the feature.

##### Inner radius variation

The inner radius (Fig. 3E), *r*_*i*_, is here defined as the median distance of every point in the path to the centre of the minimum enclosing ellipsoid. The coefficient of variation of the inner radius measures the relative dispersion of points relative to a circle. A perfect circle has a coefficient of variation equal to zero. It is here defined as 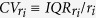, where 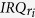 is the inter-quartile range of the inner radius.

Note: strictly speaking the coefficient of variation is defined in the literature as the standard deviation divided by the mean. Here, however, these values are replaced by the inter-quartile range and median to increase the robustness and stability against outliers.

##### Central displacement

The central displacement is the Euclidean distance of the centre of the minimum enclosing ellipsoid to the centre of the arena (Fig. 3E, “*d*”). It is an important measure to identify concentric paths with the arena.

#### Labelling of data and definition of constraints

The labelling of swimming path segments was done interactively, using a custom Graphical User Interface (GUI) written in Matlab (supplementary information). The graphical interface allowed to interactively browse through the segments, label them, and check the data clustering results. Multiple labels could be assigned to a single segment for cases in which characteristics of more than one behavioural class were found. Other labels (or *tags*), not used in the clustering process, could also be defined and assigned to segments or complete swimming paths. This was used to, for example, tag segments or trajectories of interest that would later be exported or analysed in more detail.

From the set of segments generated in the segmentation process (up to 30,000) between 5% and 12% of them were labelled (Table 2). The selection of segments to be labelled was done interactively from the complete set of segments. The custom GUI made it possible to sort segments according to different criteria, such as feature values or distance to the centre of the corresponding clusters. Various filters made it also possible to select only a subset of the segments, making it easier to identify segments that had to be labelled. One of those filters selected only isolated segments, or segments that were still not classified and did not overlap with any other successfully classified segments (see also discussion of the coverage value below). Segments that were isolated or lying on the boundaries of clusters were given priority in the labelling process.

Each pair of labels generated either a “cannot-link” (in case they differed) or “must-link” constraint (in case that they were the same). The number of constraints was therefore proportional to *N*^2^, where *N* is the number of labelled segments. Because of the large number of constraints generated in this way (more than 2 million for the full set of labels), and the resulting computational performance impact on the clustering process, constraints were defined only between relatively close points. The Euclidean distance, *d*, between two labelled data points (the same distance function employed by the clustering algorithm) was calculated and a constraint was defined only if *d* < 0.25 (feature values were all re-scaled to lie in the [0, 1] range). With this, the total number of constraints was reduced to less 10,000; however, this reduction of the number of constraints has been found to have no major impact on the clustering results.

#### Semi-supervised clustering

Semi-supervised learning methods^26^,^27^ have been the focus of much research recently (e.g.^28^,^29^). Contrary to supervised learning approaches, they are applicable to cases where large pools of data are available and labelling them all may not be possible. Whereas *unsupervised* algorithms search for structure in data without labels, *supervised* algorithms are provided with data and labels and try to infer a mapping between the two. Semi-supervised learning (SSL) can be considered an intermediate case between these two extremes. In one of the SSL formulations^30^, a pool of unlabelled data is provided together with an incomplete set of labels; the objective is again to find a suitable mapping between data and labels. Here, we chose a semi-supervised learning method since generating a fully labelled data set for the trajectory segments (between 8,000 and 30,000 for the data set analysed here) is not a viable option.

Among standard machine learning methods, we chose a clustering algorithm. The reason was that clustering makes it easy to detect new classes of behaviour, given that, in its broadest sense, clustering algorithms have as objective to group data points which are as similar as possible^31^. Therefore clustering algorithms can be used not only to classify data by finding clusters of similar segments, but also to identify new clusters with common types of behaviour. Hence, we don’t need to define *a priori* classes of behaviour but rather allow the algorithm to discover them by looking for structural similarities in segments.

Without any additional knowledge, the clustering results will depend largely on how similarity between elements is defined. There is a myriad of clustering algorithms proposed in the literature^32^. One of the first and simplest algorithms proposed is known as *k-means clustering*^33^. In this algorithm a random initial set of cluster centre points is selected followed by an interactive process, where points are moved to the nearest cluster and then new cluster centres are computed. One of the drawbacks of k-means and similar algorithms is that their outcome can vary depending on the initial set of points, which is randomly selected. If, however, additional information about the data is available, this can be used to guide the clustering process and improve the selection of the initial conditions; this scenario leads to semi-supervised clustering algorithms.

Although semi-supervised clustering algorithms have been far less studied than unsupervised clustering ones, a few such algorithms can be found in the literature. The one adopted here is known as MPCKMeans (Metric Pairwise Constrained K-Means), which was proposed by Bilenko *et al.* in 2004^34^ (more details are given in the supplementary information). This algorithm is inspired by the classical k-means algorithm, but is able to incorporate previous knowledge to guide the clustering process. This is given as a set of “must-link” and “cannot-link” constraints, instructing that certain data points should or should not be members of the same cluster. Another distinguishing feature of the algorithm is that it incorporates *metric-learning*^35^,^36^,^37^, or the ability to learn different metrics for each cluster allowing clusters to have different shapes and sizes.

As with most clustering algorithms, MPCK-means also requires a predefined number of target clusters. Determining the ideal number of clusters is a common problem in data clustering^38^,^39^; how this value was chosen here will be discussed below.

##### Two-stage clustering

Despite the fact that both “must-link” and “cannot-link” constraints can be defined between data points, only constraints of the latter type were defined during the first clustering stage. The reason for this is that the feature space is not easily separable and therefore multiple clusters can belong to one class. This means that not all must-link constraints can be satisfied and it was found that adding them at this stage had a negative impact on the clustering results. However, “must-link” type of constraints were added in the second clustering stage; this second clustering attempted to further divide larger clusters which either contained labels of more than one class or clusters which did not contain a sufficient number of labels. A detailed description of the clustering algorithm employed here, as well as comparisons with a standard single pass clustering, are given in the supplementary information.

##### Mapping clusters to classes

Labelled data was used not only to guide the clustering but also to map clusters to classes and to estimate the quality of clustering (see *Clustering validation* below).

Clusters were mapped to the class of the labelled segments within the cluster. Clusters containing labels of multiple classes, or clusters with less than a minimum amount of labels, *m*_*i*_, were marked as *undefined;* segments belonging to these clusters were discarded from further analyses. Since the clustering algorithm generates clusters with a wide range of different sizes the minimum number of required labels of cluster *i* was made dependent on the cluster size; it was defined as 
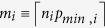
, where *n*_*i*_ is the cluster size, and 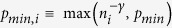; *γ* = 0.7 and *p*_*min*_ = 0.01 were adopted in our analyses.

With the definition above a larger proportion of labelled data is required in the case of smaller clusters; for larger clusters this proportion gets smaller but is always at least 1%.

#### Clustering validation, target number of clusters

The quality of the clustering was assessed by the number of classification errors, by the percentage of unclassified segments (i.e., proportion of segments belonging to one of the undefined clusters, as described in the previous Section), and by computing the *coverage* value. The coverage value is defined as the percentage of segments that were either classified (i.e., are members of one of the clusters that were mapped to classes) or which overlap with other segments that were successfully classified. In other words, it is the percentage of the trajectories that are covered by segments of known classes. Coverage was the main criterion used to evaluate the classification quality; in each classification the target was to achieve a coverage of at least 90%. The percentage of classified segments was also used to compare clustering results, but less importance was given to it compared to coverage, since its value depends strongly on factors such as the chosen segment overlap.

In order to estimate the classification error, another important measure of the quality of the clustering, a 10-fold cross-validation was used. This consisted in leaving 10% of the labelled segments for testing and not using them to define the clustering constraints. The error was calculated by dividing the number of incorrectly classified segments by the number of correct ones and taking the mean over all 10 runs. The same two-way data split was also used to tune clustering parameters, such as the target number of clusters. Since the classification performed here was targeting only one data set, and the objective was never to apply it to new data or to create a generic classifier, a two-way split was favoured over a three-way one; in doing so more labelled data was left for the clustering itself.

The percentages of unknown segments and of classification errors are not fully independent. This because rejecting more clusters will lead to less classification errors, but will also leave a larger proportion of segments unclassified. The trade-off between the two can be controlled in part by changing the minimum number of labels required to assign a cluster to a class. This is controlled by parameters *γ* and *p*_*min*_ (defined in *Mapping clusters to classes* above). Requiring more labels per cluster leads to fewer classification errors but requires more labelled data; otherwise most clusters will not have enough labels and will be left as undefined.

The percentage of undefined segments and the coverage values are also closely related, but the latter is more important as it takes into account the distribution of the classified segments. For this reason the coverage value was the main metric used to choose the appropriate target number of clusters.

A further important indicator of the quality of a classification, which can also be used to detect issues such as mixing of classes, is the confusion matrix. It counts the number of classification errors between individual classes, so that the diagonal shows the number of correct classifications and all other values show classification errors. The confusion matrix for the data here (Table 1, Supporting Information) shows that the mixing between classes was small.

#### Mapping segment classes to swimming paths

After classifying the swimming path segments, we computed the evolution of the strategies along the swimming paths. The mapping was done for discrete path intervals that depended on the segment overlap (Table 2) and took into consideration all the segments that overlap with the intervals. The corresponding segment class was selected based on the total weight of each class for the segment, 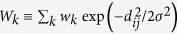, where the sum is to be taken over all segments that were mapped to class k and that intersect with the interval. The class of the interval was defined as the class with the highest total weight. In the previous expression *d*_*ij*_ is the distance from the centre of the *jth* path segment in minimum path interval units (Table 2) and *σ* was set to 4. The class weight, *w*_*k*_, was defined as *w*_*k*_ ≡ *L*_*max*_/*L*_*max,k*_, where *L*_*max,k*_ is the maximum length of consecutive segments of the *kth* class and *L*_*max*_ the maximum length among all the classes. It was made class dependent to avoid transient classes of behaviour (e.g. self-orienting) being overshadowed by other more common and usually longer lasting types of behaviour (e.g. thigmotaxis). More details of the mapping procedure are given in the supplementary information.

To produce the final results, multiple classifications (only for segments of same length) were combined. In order to combine two classifications, the classes of matching segments in both classifications were compared. Mismatches were discarded and not used in the mapping of strategies to paths. Our final results (for the analysis of stressed and non-stressed animals) were based on a combined classification with segments of 250 cm and overlaps of 70% and 90%.

### Statistics

Multi-factor testing of variance was done using a Friedman test^40^, a non-parametric test that is well suited for data that is not normally distributed. Besides this important characteristic of our data, the Friedman test is also a *matched* test, and can control for experimental variability among subjects. In our case, the same animals were analysed over a set of 12 trials; the variability between different trials, that affects all animals, is not taken into account in the Friedman test.

The p-values shown in our analyses answer the question: if the different treatments (control vs. stress) are identical, what is the chance that a random sampling would result in the distribution of values (or *ranks*, as used by the Friedman test) as far apart as observed? Small p-values (<0.05 in our analyses) lead us to discard the null hypotheses that the results are identical and differences are only due to random sampling.

## Additional Information

**How to cite this article**: Gehring, T. V. *et al.* Detailed classification of swimming paths in the Morris Water Maze: multiple strategies within one trial. *Sci. Rep.* **5**, 14562; doi: 10.1038/srep14562 (2015).

## Supplementary Material

Supplementary Information

## Figures and Tables

**Table 1:** Features used for the classification of swimming path segments.

Feature	Definition
Median distance to centre	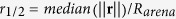 , where **r** is the set of vectors to the centre of the arena for each point of the segment
Interquartile range of the distance to centre	(*r*_75_ − *r*_25_)/*R*_*arena*_ where *r*_25_(*r*_75_) is the first (third) quartile of the distance to the centre (Fig. 3B)
Focus	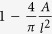 *, l* = segment length, *A* = area covering the segment (Fig. 3C)
Target proximity	Percentage of path lying within an area centred on the platform and with radius equal to 6 times the platform radius (Fig. 3D area *T*_1_)
Eccentricity	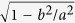 (Fig. 3C)
Maximum loop length	Length of the longest self-intersecting loop in the trajectory divided by the segment length (see Fig. 3D, loop “*l*”)
Inner radius variation	(*r*_*i*,75_ − *r*_*i*,25_)/*r*_*i*_, where *r*_*i*_ is the inner radius, or median distance to the centre of the surrounding ellipse (Fig. 3E)
Central displacement	Distance from the centre of the surrounding ellipse to the centre of the platform divided by the arena radius (Fig. 3E, “*d*”)

All features are dimensionless and only weakly correlated with the choice of segment length as to improve the classification robustness against segment length variations.

**Table 2:** Parameters and results for three different classifications with variable segment lengths and overlaps.

	Classification 1	Classification 2	Classification 3
Segment length	300 cm	250 cm	250 cm
Segment overlap	70%	70%	90%
Minimum path interval	90 cm	75 cm	25 cm
Segments	8,847	10,388	29,476
Labels	989	1,301	1,605
Clustering constraints	6,464	7,390	6,599
Clusters (first stage)	37	35	75
Clusters (final number)	125	139	200
Unclassified segments	18.8%	20.0%	28.2%
Classification errors	0.51%	0.62%	0.19%
Coverage	94.1%	94.9%	97.3%
Agreement with 1	–	77.8%	72.9%
Agreement with 2	77.8%	–	89.4%
Agreement with 3	72.9%	89.4%	–

Different numbers of target clusters in the first clustering stage were used and those giving the best results were chosen. The clustering algorithm includes a second stage where larger clusters with mixed labels are further sub-divided. The coverage value indicates the percentage of the swimming paths that are covered by segments of known classes. The agreement between two classifications was computed by comparing the classes of the corresponding segments. The classifications do not fully agree because of the transition segments between two classes, which can be mapped to either of the classes; this, however, does not have a major impact in the classification results.

**Table 3:** Transition probabilities of strategies within trials for the control (left) and stress (right) group of animals.

	TT	IC	SC	FS	CR	SO	SS	ST
TT	–	0.505	0.035	**0.234**	0.022	0.036	0.153	0.015
IC	0.354	–	0.055	0.121	0.128	0.134	0.172	0.036
SC	0.220	0.307	–	0.098	0.033	0.081	0.168	0.094
FS	**0.449**	0.253	0.015	–	0.051	0.045	0.154	0.033
CR	0	0.302	0	0	–	**0.372**	**0.326**	0
SO	0.276	0.247	0.108	0.121	0.082	–	0.089	0.078
SS	0.322	0.267	0.060	0.040	0.139	0.057	–	0.116
ST	0.165	0.349	0.083	0.083	0	**0.128**	0.202	–

	TT	IC	SC	FS	CR	SO	SS	ST
TT	–	0.538	0.017	0.134	0.054	0.075	0.167	0.020
IC	**0.515**	–	0.054	0.108	0.073	0.071	0.156	0.023
SC	0.250	0.295	–	0.065	0.095	0.131	0.110	0.053
FS	0.268	**0.421**	0.025	–	0.101	0.046	0.117	0.021
CR	**0.322**	0.229	0.059	0.024	–	0.094	0.146	**0.126**
SO	0.313	**0.357**	0.057	0.042	0.061	–	0.146	0.024
SS	0.329	**0.364**	0.043	0.055	0.123	0.040	–	0.046
ST	**0.281**	0.302	0.024	0.132	0.059	0.021	0.181	–

Rows and columns indicate the starting and ending strategies respectively. Row values (for the same starting strategy) are normalised. Bold values indicate the most significant differences between the two groups. Results show a higher tendency for stressed vs non-stressed animals to move to less efficient strategies (thigmotaxis, incursion, and scanning - the ones where chances of finding the platform are reduced); this is in agreement with results from Fig. 5. *TT* = thigmotaxis, *IC* = incursion, *SC* = scanning, *FS* = focused search, *CR* = chaining reaction, *SO* = self orienting, *SS* = scanning surrounding, *ST* = scanning target.
