# Supplementary material for: Detailed classification of swimming paths in the Morris Water Maze: multiple strategies within one trial
Source: Sci Rep. 2015 Oct 1;5:14562. doi: 10.1038/srep14562 (PMC4589698; doi:10.1038/srep14562)
Supplement: Supplementary Information [file srep14562-s1.pdf]

Supporting Information Material

Detailed classification of swimming paths in the Morris Water Maze: multiple strategies within one trial

T.V. Gehring, G. Luksys , C. Sandi, E. Vasilaki

Contents

1 Classification method **2**
1.1 Details of the segmentation process 3
1.2 Computation of features 3
1.3 Labelling of segments 3
1.4 Two-stage clustering algorithm description 3
1.4.1 MPCKMeans algorithm description 6
1.5 Clustering validation: confusion matrix 6
1.6 Mapping segment classes to trajectories 6

2 Software tools **9**

3 Data calibration **9**

1 Classification method

The classification procedure consisted of the following steps (Figure 1):

1. Segmentation of trajectories to overlapping segments of fixed length;
2. Computation of feature values for each segment;
3. Labelling of stereotypical segments for each segment class of interest;
4. Clustering and mapping of clusters to classes using the labelled data;
5. Evaluation of clustering performance. This is done by computing three quantities: the coverage, or percentage of swimming paths covered by successfully classified segments, the percentage of segments that could not be classified (because they belong either to a cluster with an insufficient number of labels or with labels of multiple classes), and the cross-validation error (average percentage of the test data that was assigned to a cluster of the wrong class).
6. Steps 3-5 are repeated until an acceptable clustering quality is found;
7. Computation of the distribution of behavioural classes for each trajectory;

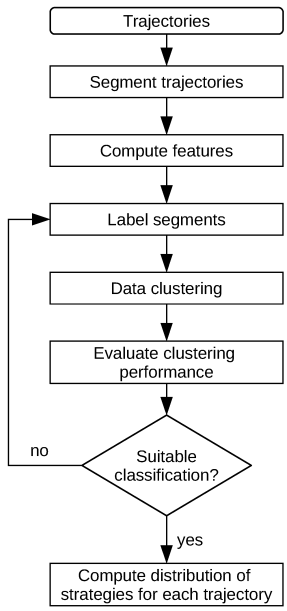

Figure 1: Steps of the classification procedure

In the next sub-sections more details of some of the aspects of the classification method are presented.

1.1 Details of the segmentation process

Trajectories were split into segments of length d, where segment i is defined as the set of points of the recorded trajectory lying in the interval $[l_i, l_i + d]$. The segmentation process generates segments that significantly overlap to reduce the classification variance due to unfavourable segmentations. For the analysis performed here overlaps of 70% and 90% were adopted. The number of segments for a trajectory of length L, segment length d, and overlap α is $N = \lceil (L/d - 1).(1 - \alpha)^{-1} \rceil$, where $\lceil .. \rceil$ is the ceiling function. Trajectories shorter than the segment length are mapped to a single segment. The starting point of segment i, $1 \leq i \leq N$, is $l_i = d \cdot (1 - \alpha)(i - 1)$.

1.2 Computation of features

Features of each segment were calculated as described in main text, Methods.

1.3 Labelling of segments

The labelling of segments was done iteractively, as described in the main text, Methods.

1.4 Two-stage clustering algorithm description

Experiments showed that the 2-stage clustering algorithm adopted here improves the clustering performance considerably (Fig. 2). The algorithm is detailed in Algorithm 1. In the first step, the algorithm clusters the data into the target number of clusters using only "cannot-link" types of constraints. Clusters are then mapped to label classes (how this mapping is done is discussed in the next Section) and clusters which could not be unambiguously mapped to a class are sub-divided further by another clustering step. It this second clustering step each cluster that could not be mapped to a single class in the first step is split once more, this time, however, using both must-link and cannot-link constraints; multiple target number of clusters (from 2 up to two times the number of different classes in the original cluster) are also tried in succession. The first successful sub-partitioning, or the one with the smallest target number of clusters, is then chosen (a partitioning is considered successful if at least one of the sub-clusters could be mapped to a single class).

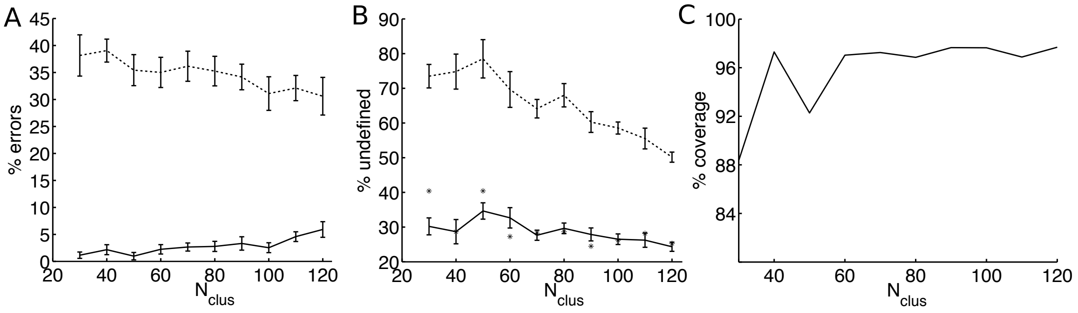

Figure 2: **A-B:** Impact of the number of clusters on the clustering performance for a set of 29,476 segments (Classification 3 in Table 2 in the main text). **A** Percentage of classification errors (percentage of labelled segments mapped to the wrong class); **B** Percentage of segments belonging to clusters that could not be mapped unambiguously to a single class; Error bars represent the 95% CI of a ten-fold cross validation over a set of 1,605 labels. *Continuous lines*: two-stage clustering. *Dashed lines*: single stage clustering. The results show that the second clustering stage leads both to significantly less classification errors and a smaller percentage of unclassified segments. Asterisks in the middle plot mark the results when using the full set of labels. **C** Percentage of the full swimming paths that are covered by at least one segment of a known class. The target number of clusters, N_{clus}, was chosen so that the coverage value is as high as possible while still having a low number of classification errors.

As Fig. 2 shows, the two-stage clustering shows significantly better results than a single clustering stage. The two-stage algorithm also leads to less variance of the final results over different target number of clusters.

Data: feature vectors $\mathbf{x}_i$, $1 \leq i \leq N$
labels $\{L, L_2, ..., L_N\}$ with $(L_i = \emptyset \vee L_i = \{l_{i1}, ..., l_{in_i}\})$
target number of clusters k
maximum constraint distance d_{max}
Result: clusters $\{C_1, C_2, ... , C_l\}$, $C_i = \{c_{i1}, c_{i2}, ... , c_{i\,n_i}\}$
$1 \leq c_{ij} \leq N$; c_{ij} = index of jth element of ith cluster
cluster labels $\{\mathcal{L}_1, \mathcal{L}_2, ... , \mathcal{L}_l\}$
`/* initialization */`
$\mathcal{M} \leftarrow \emptyset$, $\mathcal{C} \leftarrow \emptyset$; `/* must/cannot-link constraints */`
`/* creation of constraints */`
foreach $\{i, j\} \,|\, L_i \neq \emptyset \wedge L_j \neq \emptyset \wedge |\mathbf{x}_i - \mathbf{x}_j| < d_{max}$ **do**
  if $L_i \cap L_j \neq \emptyset$ **then**
    $\mathcal{M} \leftarrow \mathcal{M} \cup \{i, j\}$; `/* create must-link constraint */`
  else
    $\mathcal{C} \leftarrow \mathcal{C} \cup \{i, j\}$; `/* create cannot-link constraint */`
  end
end
`/* main algorithm */`
cluster data into k clusters $C_1, C_2, ..., C_k$ using constraints $\mathcal{C}$
foreach *cluster* C_l **do**
  if $C_l = \emptyset$ **then**
    discard C_l and move to next cluster
  end
  map cluster C_l to label $\mathcal{L}_l$
  $m_l = |M_l|$, $M_l = (L_{l_1} \cap L_{l_2} \cap ... L_{l_n} | \{l_1, ..., l_n\} \in C_l)$;
  `/* number of distinct labels in` C_l `*/`
  if $m_l \geq 1 \wedge \mathcal{L}_l = \emptyset$ **then**
    $k'_l \leftarrow \max(m_l, 2)$; `/* number of sub-clusters */`
1   cluster C_l into k'_l sub-clusters $C'_{l1}, ... , C'_{l|k'_l}$ using constraints $\mathcal{C} \cup \mathcal{M}$
    map clusters $C'_{l1}, ..., C'_{l|k'_l}$ to classes $\mathcal{L}'_{l1}, ... , \mathcal{L}'_{l|k'_l}$
    if $\mathcal{L}'_{l|i} \neq \emptyset$ *for any* $1 \leq i \leq k'_l$ **then**
      $C_l \leftarrow \{C'_{l1}, ... , C'_{l|k'_l}\}$; `/* accept sub-clustering */`
      $\mathcal{L}_l \leftarrow \{\mathcal{L}'_{l1}, ... , \mathcal{L}'_{l|k'_l}\}$
    else
      if $k'_l < 2\,m_l$ **then**
        $k'_l \leftarrow k'_l + 1$; `/* increase number of sub-clusters */`
        jump to 1
      end
    end
  end
end

Algorithm 1: Two-stage clustering. Steps 1 and 2 define the set of constraints. The main clustering, using only "cannot-link" constraints, is done at step 3. Steps 4 describes how clusters that were not mapped to a class, because they contained multiple label classes or an insufficient number of labels of one class, are sub-divided. The sub-division process attempts to break down the cluster into an increasing number of smaller ones until the smaller clusters can be mapped to classes (in which case the larger cluster is replaced by the smaller ones - step 4h) or an upper limit of sub-clusters is reached.

1.4.1 MPCKMeans algorithm description

The MPCKMeans clustering algorithm uses the provided constraints to build an objective function (Equation 1) that is then iteratively minimised.

$$\mathcal{J} = \sum_{\mathbf{x_i} \in \mathcal{X}} \|\mathbf{x}_\mathrm{i} - \mu_{\mathrm{l_i}}\|^2 + \sum_{(\mathbf{x_i},\, \mathbf{x_j}) \in \mathcal{M}} \mathrm{w_{ij}} [\mathrm{l_i} \neq \mathrm{l_j}] + \sum_{(\mathbf{x_i},\, \mathbf{x_j}) \in \mathcal{C}} \overline{\mathrm{w}}_{\mathrm{ij}} [\mathrm{l_i} = \mathrm{l_j}] \tag{1}$$

In Equation (1) $\mathbf{x_i}$ is the feature value vector of the ith element, whereas the first term is the cost of assigning element $\mathbf{x}_i$ to cluster $\mathcal{X}_{\mathrm{l_i}}$ with centroid μ_{l_i}. Metric learning changes this term to allow for non-uniform weights for each cluster (for details see [1]). The second and third terms are costs of violating the pairwise must-link ($(\mathbf{x}_i, \mathbf{x}_j) \in \mathcal{M}$) and cannot-link ($(\mathbf{x}_i, \mathbf{x}_j) \in C$) constraints, with penalties w_{ij} and $\overline{w}_{ij}$ respectively. Because violation of constraints is allowed, they are known as *soft constraints* and the minimisation process is guaranteed to converge only to a local, and not global, minimum.

Here we use the standard MPCKMeans implementation[1]. The code is written in Java and is provided as part of the WekaUT library, a modified version of the popular Weka (Waikato Environment for Knowledge Analysis) machine learning library [2]. The Java code was integrated directly into Matlab and all algorithm options (such as the metric function and constraint weights) were left at their defaults.

1.5 Clustering validation: confusion matrix

The confusion matrix can be a valuable tool to identify classes that are not being well separated. Figure 1 shows the confusion matrix for the data at hand where a 10-fold cross-validation was performed. As can be seen the number of classification errors is small and relatively spread out through the matrix, so the classification performance is approximately homogeneous among the classes.

Table 1: Confusion matrix for the classification of the segments. Values are the total number of miss-classifications for a 10-fold cross validation of the clustering algorithm (i.e. 10 runs using 10% of the values each time for validation). Values in the diagonal show the number of correct classification for this class. The confusion matrix can be used to identify classes that are not well separated in the classification process (non-diagonal values different than zero)

	TT	IC	SC	FS	CR	SO	SS	TS
Thigmotaxis (TT)	267	14	0	0	0	0	2	0
Incursion (IC)	8	331	3	0	4	0	4	2
Scanning (SC)	0	3	144	0	0	0	2	0
Focused search (FS)	0	0	1	72	0	0	0	1
Chaining response (CR)	0	8	0	0	90	0	1	0
Self orienting (SO)	0	0	0	0	0	61	0	0
Scanning surroundings (SS)	2	11	0	0	3	0	252	0
Target scanning (TS)	0	5	0	0	0	0	2	73

1.6 Mapping segment classes to trajectories

The mapping of classes of behaviourto swimming paths was done for each minimum path interval (which dependend on the segmentation parameters). The choide of class for each interval took into consideration all the segment classes that intersected a given interval. The corresponding segment class was computed from the following expression:

[1]available at http://www.cs.utexas.edu/users/ml/risc/code/

$$\mathcal{C}_{T_i} \equiv \arg\max_{\mathrm{c_k}} \sum_{\substack{\mathrm{S_j \in c_k} \\ T_i \cap S_j \neq \emptyset}} w_k \exp\left(-d_{ij}^2/2\sigma^2\right) \tag{2}$$

where T_i is the ith interval, d_{ij} is the distance from the centre of the jth path segment, S_j, to the interval, $c_k$is the kth segment class, and w_k is a class weight normalised so that $\sum w_k = 1$. The sum is to be taken over all segments intersecting with the path interval T_i and which were mapped to the class c_k. The above expression effectively gives a higher weight to the central parts of trajectory segments and to certain behavioural classes. In the equation above the distances dij between segments and path intervals were computed in minimum path interval units (see Table 2 in main text). The parameter σ, which controls how much segments influence the choice of segment classes over the swimming paths, was set to $\sigma = 4$ and the class weight, w_k, was defined as

$$w_k \equiv \frac{L_{max}}{L_{max,k}} \tag{3}$$

where $L_{max,k}$ and L_{max} are the maximum length of continuous segments of class k and of all homogeneous segments, respectively.

The weight was made class dependent and inversely proportional to the maximum length of segments of the class so that transient classes, or classes which tend to be shorter, do not get overshadowed by more common ones. Figure shows one example using constant class weights ($w_k = 1$) and using the above definition. As can be seen in the former case the self-orienting and focused search segments do not get detected because of the larger influence of surrounding classes (incursion/thigmotaxis).

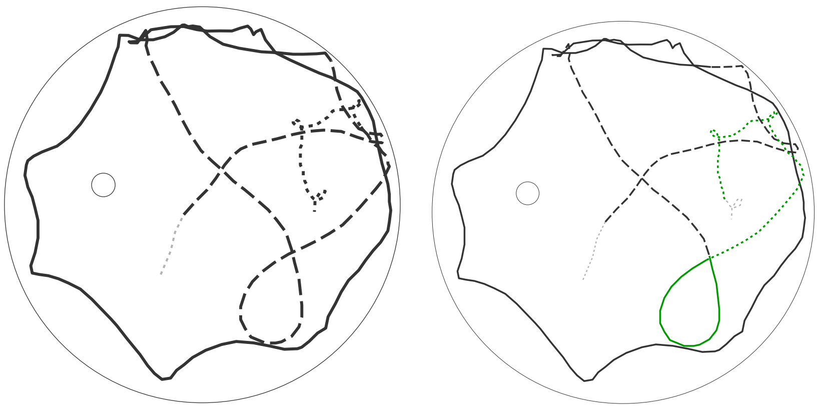

Figure 3: Detailed classification of one trajectory. *Left*: classification results using constant class weights. *Right*: results using weights as defined in Eq. 3. As can be seen in the former case the self-orienting loop (continuous green line) and focused search segments (dotted green line) did not get detected and were replaced by neighboring classes (incursion, dashed black lines, and scanning, dotted black lines, respectively).

Table 2 shows the average and maximum lengths of segments of each class using first $w_k = 1$ and then the definition above. An example of a trajectory classified with constant and variable weights is shown in the Supplemental Material.

Method validation

Figure 4 shows the manual classification results of the full trajectories. The manual labelling included only four major behavioural classes: thigmotaxis, target scanning, incursion, and scanning.

Table 2: Mean and maximum length of consecutive segments of each class for the 250 cm / 90% overlap classification (see Table 2 in the main text) with constant weights ($w_k = const.$ in Eq. 2) and after adopting differentiated weights for minor and major classes (Eq. 3).

	Mean (old)	*Max (old)*	*Weight*	*Mean (new)*	*Max (new)*
Thigmotaxis	359.3	3,000	1.0	328.6	3,000
Incursion	194.5	1,225	2.4	191.2	1,200
Scanning	118.5	850	3.5	105.7	725
Scanning surroundings	145.2	675	4.4	155.5	650
Focused search	107.4	550	5.4	125.5	575
Self orienting	105.1	375	8.0	139.1	400
Target scanning	123.2	350	8.6	129.6	375
Chaining response	97.3	300	10.0	139.1	400

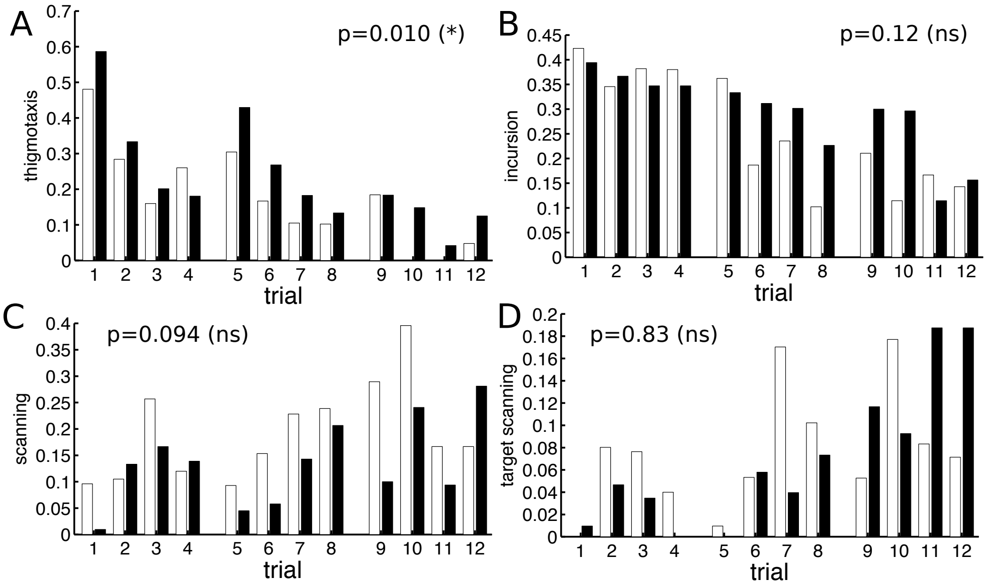

Figure 4: Results of a manual classification of complete swimming paths. Labels corresponding to four behavioural classes (thigmotaxis, incursion, scanning, and target scanning / plots A-D respectively; see main text, Figure 1, for a description of the classes) were assigned to swimming paths depending on the types of behaviour that were identified. Multiple behavioural classes could be assigned to each swimming path; the percentage corresponding to each class was calculated as an average of the number of identified classes in each swimming path. *White bars*: control group; *Black bars*: stress group. Both groups were compared over the complete set of trials using a Friedman test. Plot A shows that stressed animals have a clear tendency to look for wall contact more often (thigmotaxis).

Full classification results

Figure 5 shows the detailed classification of full set of swimming paths from the first six trials, both for the control (top) and the stress (bottom) group (27 animals each). Each bar represents a full trial (up to 90 seconds) and shows changes in exploration strategies over the trial.

2 Software tools

In order to classify the trajectory segments custom code and a custom Graphical User Interface (GUI) were developed in Matlab (Figure 6). The GUI made it possible to interactively label segments, see the classification results and identify problems such as clusters which insufficient number of labels.

Following main features were implemented in the GUI:

- Browsing and labelling of swimming paths and path segments; available classes are defined in the main configuration file used by the code. Multiple labels could be assigned to a swimming path and multiple label sets (for different classification) can be stored;
- Running the clustering algorithm for different target number of clusters, with or without cross-validation (to estimate the classification error);
- Highlighting of classes that each segment was mapped to; displaying statistics such as number of labelled segments of each class and percentage of classification errors;
- Filtering swimming paths showing only the ones that fulfil a specific criteria such as segments that were assigned to one particular clusters, ones which where wrongly classified or which are isolated (i.e. do not have neighbouring segments of a known class), or which were mapped to a class which is different from the one which they were mapped to in another reference classification (allowing to compare two classifications with different segment lengths or overlaps, for example);
- Sorting of segments according to one feature value, a combination of all features (using a distance function), or according to the maximum distance to the centre of the corresponding clusters (useful to detect elements that lie close to the boundaries of the clusters);

Besides the custom GUI many other classes and support code was developed to analyse the swimming paths. This included, for example, import routines for swimming paths stored as text files exported by EthoVision, routines to perform the segmentation of swimming paths, code for computing the various different features for each segment, and code to generate the constraints based on a set of segment labels and run the clustering algorithm.

3 Data calibration

Swimming paths of rats were recording using an object tracking software (EthoVision [3], version 3.1). Due to the relatively close range of the camera and the lens system used the recorded trajectories have to be first calibrated.

We calibrated the trajectories by using the trajectories as show on screen in the tracking software, which has its own built-in calibration method, as reference. To extract the reference points for from software its playback capability showing a swimming path step by step as recorded was used; screenshots were named as to give an indication about the recording time at which they were taken. One example screenshot is shown in Figure 7. The current position of the animal is shown as a black (dark-grey) square; this feature was used to automatically detect the position from the screenshots using imaging processing routines. As reference the outer (yellow) circle was used. The so extracted coordinates were then compared to the ones exported from the software, using the sample time information as shown in the screenshots (seen on the bottom right).

The pairs of real and exported coordinates were then used to compute a pair of error functions for x and y directions. These functions used a linear interpolation of the differences for estimating the correction for the given coordinate along the trajectory

$$d_x(x) = \delta x_i + (\frac{x - x_i}{x_{i+1} - x_i})(\delta x_{i+1} - \delta x_i), \quad x_i \leq x < x_{i+1} \tag{4}$$

$$d_y(x) = \delta y_i + (\frac{y - y_i}{y_{i+1} - y_i})(\delta y_{i+1} - \delta y_i), \quad y_i \leq y < y_{i+1} \tag{5}$$

where δx_i and δy_i, $i = 1, 2, ..., N$, are the sorted differences found between the N extracted points from the trajectories and the respective exported coordinates. Figure 8 shows the two sets of error functions used to calibrate the data at hand.

The error functions were then used to correct the complete set of trajectories. Figure 9 shows an example of a distorted trajectory as exported by the tracking software and the same trajectory after applying the correction.

The calibration was validated by using a (ten-fold stratified) cross-validation measuring the error of the interpolated function with calibration points not used in the interpolation (Figure 10). The results show the average error (x and y axis) for different number of calibration points; as can be seen the improvements are only minimal when using more than 500 calibration points (representing 7-8 trajectories in the data used here).

For the final data calibration the full set of data points was used, 1654 for each coordinate for the first set and 1195 for the second.

References

[1] Bilenko, M., Basu, S. & Mooney, R. J. Integrating constraints and metric learning in semi-supervised clustering. *Twenty-first international conference on Machine learning - ICML '04* 11 (2004).

[2] Holmes, G., Donkin, A. & Witten, I. Weka: A machine learning workbench. *Intelligent Information Systems. Proceedings of the Australian New Zealnd Intelligent Information Systems Conference* 357–361 (1994).

[3] Noldus, L. P. J. J., Spink, A. J. & Tegelenbosch, R. a. J. EthoVision: A versatile video tracking system for automation of behavioral experiments. *Behavior Research Methods, Instruments, & Computers* **33**, 398–414 (2001).

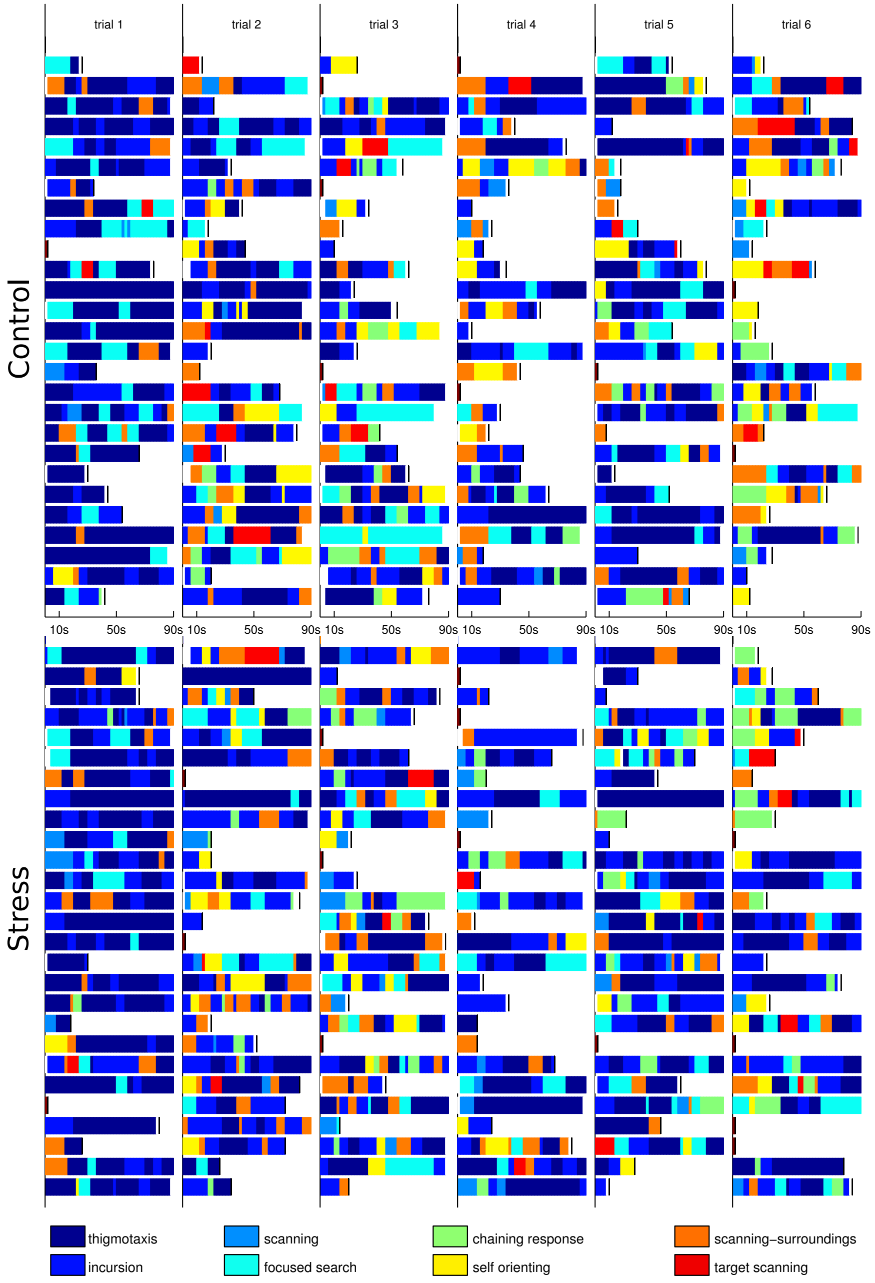

Figure 5: Detailed classification of swimming paths for the first 6 trials and for all the animals. Top: control group. Bottom: stress group. Bars show the choice of strategies over a 90 second trial. Short paths, where the animal found the platform directly, and which were not segmented, are marked in dark red. White boxes indicate segments with behaviour not falling into any of the classes and which could not be categorised.

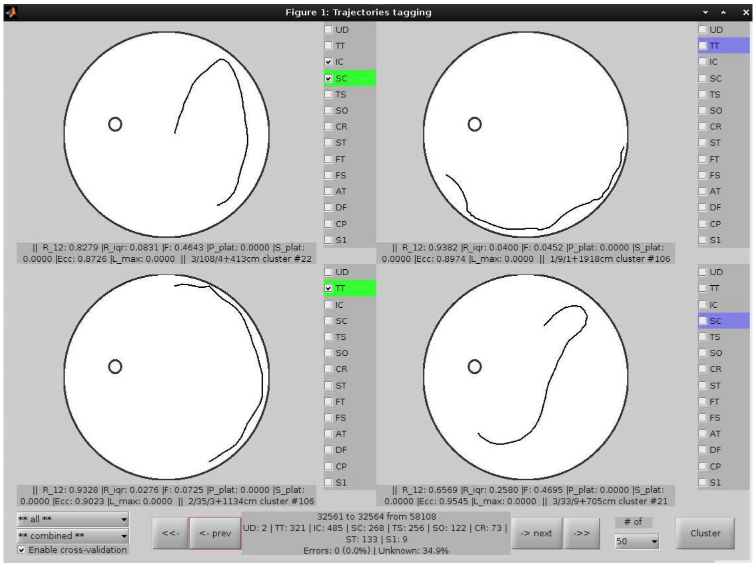

Figure 6: Graphical User Interface used to classify trajectory segments

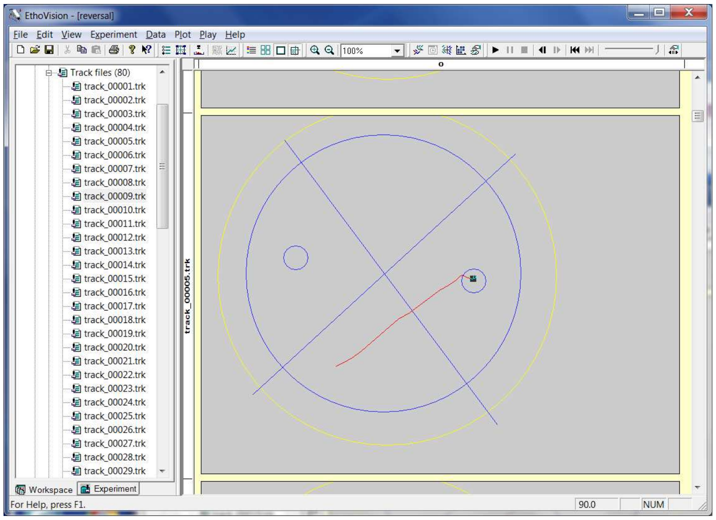

Figure 7: Snapshot from the object tracking software (EthoVision) showing one trajectory segment. Current position of the animal is shown by the black square. The position of the black square relative to the yellow circle was used to automatically estimate the current position of the animal (using image processing algorithms).

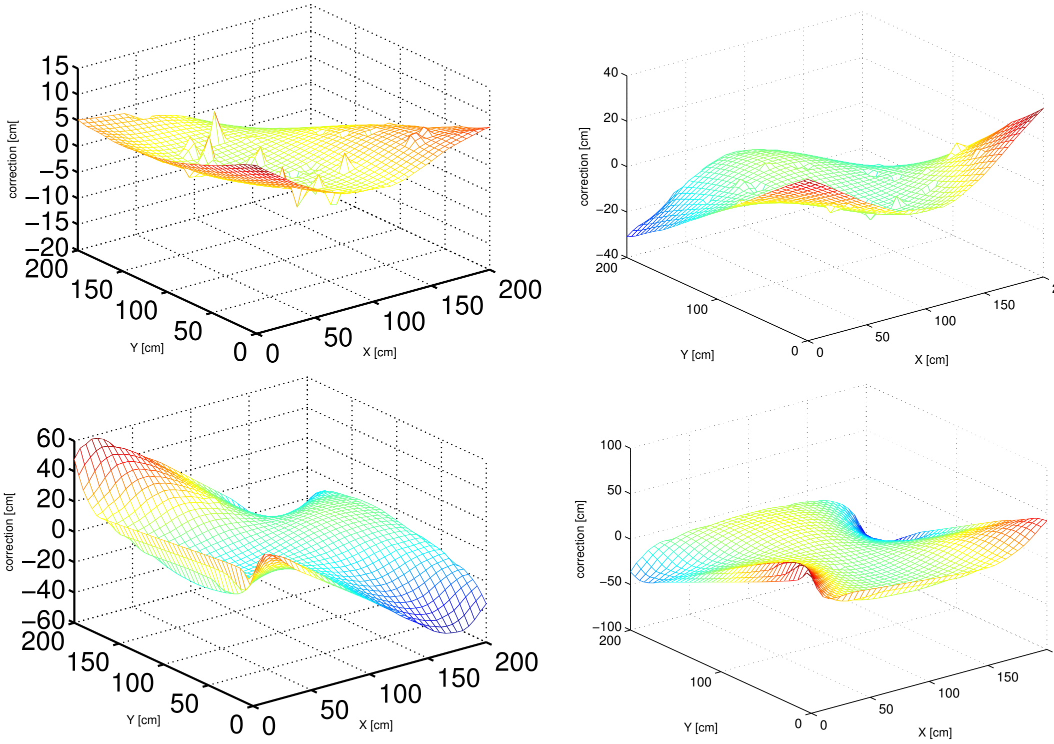

Figure 8: Calibration functions. *Left:* x axis correction. *Right:* y axis correction.

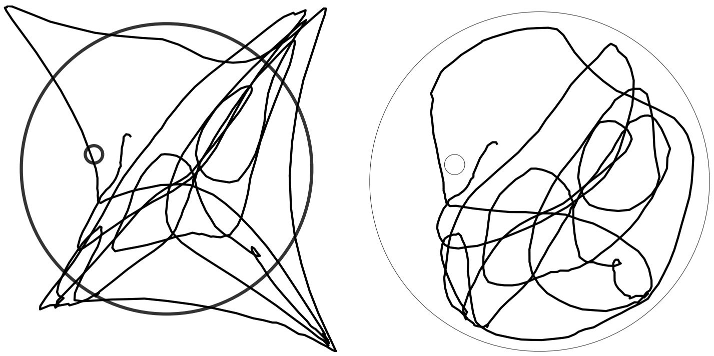

Figure 9: Example of data calibration applied to one trajectory. *Left*: original trajectory as exported by the tracking software. *Right:* corrected trajectory that closely matches the trajectory shown by the tracking software on the screen.

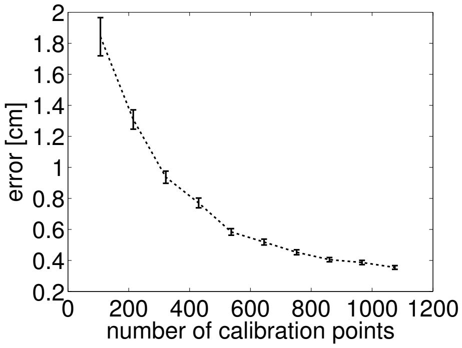

Figure 10: Calibration error as a function of the number of calibration points. The error represents the distances (in the x or y axes) between corrected coordinates and points as extracted from snapshots taking from the recording software. Only minor improvements are observed after around 500 calibration points. Error bars represent the 95% CI.
